# Heterogeneous determinants of falling birth rates in the Western Pacific

**DOI:** 10.1016/j.lanwpc.2025.101785

**Published:** 2025-12-26

**Authors:** The Lancet Regional Health – Western Pacific

Fertility decline is a global trend but particularly a concern in the Western Pacific region. Since 2021, the total fertility rates in many parts of Asia have already fallen below 2.1 per woman, the replacement level needed to prevent a population from shrinking. In this December issue, we present a series on Low Birth Rate in the Asia-Pacific discussing the heterogeneous determinants of fertility issues in the region. Falling birth rate poses a universal threat to all societies as it is linked with shrinking workforces and economic growth, as well as challenges in sustaining aging populations. It is thus a pressing priority for all policy makers managing this emerging demographic transition.

The Western Pacific region shows the highest prevalence of infertility at 23.2%, compared with the global average of 17.5%. Infertility is defined as the inability to achieve pregnancy after at least 12 months of regular unprotected intercourse. In modernised societies, infertility is never caused by a single factor or single sex but a complex interplay of physiological factors. The review by Yang and colleagues highlights that women's infertility is shaped by multiple lifestyle factors such as the inadequacy of “pro-fertility” nutrients, sedentary behaviours, chronic stress from intensive work cultures, as well as exposure to environmental pollutants following decades of industrialisation. Although female factors are more frequently studied, male infertility is an equally important topic yet too often overlooked, particularly in patriarchal cultures. Skerrett-Byrne and colleagues emphasise that men are susceptible to biological and environmental risk factors that compromise sperm health just as much as they affect female reproductive health. Between 1973 and 2018, sperm count drastically fell by 51.6% globally, with pollutants and modern lifestyle changes suspected as key drivers. Male factors are implicated in half of all infertility cases, and 20% of cases are solely attributable to men. This underscores the urgent need to go beyond a female-centric lens and broaden the narrative around infertility.

Partners of both sexes and healthcare providers should therefore set aside gender biases and address the root causes together given the multifactorial nature of infertility, yet gaps remain as we translate the knowns into concrete steps. As people become more aware of the risk factors, we still lack evidence-based advice to comprehensively guide new nutritional recommendations. Some literature has suggested possible fertility benefits of vitamin D and polyunsaturated fatty acids among people with polycystic ovary syndrome and obesity, but few authorities give definitive advice on supplementation or recommended intake levels beyond the more studied nutrients such as folate. This reflects limitations in the evidence base owing to small-scale inconsistent findings, lack of studies in the Western Pacific population, and incomplete understanding of mechanisms of action, which impede consensus. Similarly, not all couples with suspected infertility in the Western Pacific receive standardised evidence-based blood tests and scans in the first point-of-contact to expedite diagnosis and referrals. Even so, the affordability of advanced fertility treatment remains a barrier to many. In some Asian countries, the costs of assisted reproductive technology are up to 200% of GDP per capita, and countries with relatively lower costs are likely to have government financing assistance and regulations.

In November, 2025, WHO issued the first-ever global guideline for infertility, which clearly outlines the diagnostic and treatment pathway to address both male and female factors, and provides cost-effective options at every stage. Researchers in Australia also recently co-developed the first male infertility guideline to promote standardised simultaneous evaluation for men as women seek care. These frameworks are important steps to reduce barriers and time to fertility solutions, and similar models are urgently needed to adapt across the region.

The other side of low birth rate is the diminishing wish to have children, independent of fertility health. The review by Yeung and colleagues summarises the social determinants of reduced willingness to give birth including the cost of living and the financial confidence to secure children's success. Modernisation of societies has also driven ideological change such as greater individualism and women's participation in the workforce following more equitable education opportunities in both sexes, and the increased life choices contribute to delayed marriage and childbearing. In response, pronatalist measures were introduced by Asian governments including those enacted anti-natalist policies in the past decades. China abolished its One Child Policy in 2021, cash incentives per each baby born and work-life balance arrangements are offered in Japan, and Hong Kong offers tax relief for couples receiving assisted reproductive technology. However, one could argue if the investments suffice to reverse the trend. For example, the continuum of care from conception, antenatal care, to parenthood after birth requires comprehensive support, and cash incentives alone might not offset the medical cost, psychological impact, and long-term economic burden of raising children. In east Asian cultures, workplace discrimination against employees who have family plans is also an issue linked to productivity loss despite the legal entitlement to parental leave. Target-driven policies can be ineffective if they do not encourage behavioural change to respect each family decision. Low birth rate might just reflect the overall social preference, which is after all shaped by the broader culture.

Countries should account for the heterogeneity of drivers as they adapt to the transition toward low-fertility societies. In line with the UN's principle of reproductive rights being “the basic rights that all couples and individuals to decide freely and responsibly the number and spacing of their children”, we call for a full-spectrum support for all people of reproductive age—we must respect individual's choices to plan their families at their own pace while removing unfavourable cultural and structural barriers wherever possible. For families who desire children, however, a continuum of accessible, evidence-based care with trustable information should be promoted from diagnosis to treatment, such that families feel well-supported throughout the reproductive journey.

